# Primary hepatic malignant fibrous histiocytoma mimicking hepatocellular carcinoma: A report of two cases

**DOI:** 10.3892/ol.2014.2483

**Published:** 2014-08-27

**Authors:** JINGHUI DONG, WEIMIN AN, WEI MA, XIAODONG GUO, YUANZHI GAO, CHANGCHUN LIU, YONGWU LI

**Affiliations:** 1 Medical Imaging Center, The 302nd Hospital, Beijing 100039, P.R. China; 2 Department of Pathology, The 302nd Hospital, Beijing 100039, P.R. China

**Keywords:** malignant fibrous histiocytoma, histology, magnetic resonance imaging

## Abstract

Malignant fibrous histiocytoma (MFH) is a tumor that occurs throughout the body as a relatively uncommon entity. The current study presents two cases of primary malignant fibrous histiocytoma of the liver. The first case was of a 67-year-old male who exhibited no symptoms or abnormal physical signs, and in whom the lesion was found by ultrasound examination during a routine physical examination. The second case was of a 35-year-old male who presented with persistent malaise, weight loss and intermittent right upper quadrant pain. The presence of liver cirrhosis due to hepatitis B virus, which was identified 10 years previously, and the clinical appearance caused MFH to appear as hepatocellular carcinoma at the time of the initial diagnosis. Abdominal magnetic resonance imaging scans were the main tools of diagnosis, but the MFH mimicked hepatocellular carcinoma due to the similar morphological characteristics, the rare occurrence of MFH and the underlying diseases of the liver. The first patient underwent a complete resection and recovered well, while the second patient underwent palliative treatment due to the large size of the tumor and the obstructive emboli in the portal vein. The diagnoses of the tumors were confirmed as MFH by histopathology and immunohistochemistry.

## Introduction

Malignant fibrous histiocytoma (MFH), also known as undifferentiated pleomorphic sarcoma, is the most common type of sarcoma in adults aged ≥50 years (1). Although MFH can occur almost anywhere, including in the bone, due to its mesenchymal origin, it normally occurs in the deep planes of proximal extremities, in the retroperitoneum and in the torso (1–5). Retroperitoneal involvement is relatively uncommon however, and the presence of MFH in a visceral organ is exceedingly rare. Primary MFH of the liver is an unusual condition, accounting for <1% of primary hepatic malignancies (2). Two cases with primary MFH of the liver occurred in The 302nd Hospital (Beijing, China). The present study reports those two novel cases, with particular emphasis on the magnetic resonance imaging (MRI) findings. Written informed consent was obtained from both patients.

## Case report

The present study reports two cases of primary hepatic MFH, with analysis of the ultrasound and MRI findings. The first case was of a 67-year-old male who had no symptoms or abnormal physical signs on routine physical examination. Other possible significant laboratory abnormalities, which included changes in serum transaminase (aspartate transaminase, 20 units/l; normal range, 8–40 units/l; alanine transaminase, 32 units/l; normal range, 5–40 units/l), serum bilirubin (total bilirubin, 18.6 μmol/l; normal range, 3.4–20.5 μmol/l; direct bilirubin, 6.8 μmol/l; normal range, 0–6.8 μmol/l) and serum α-fetoprotein (2.0 ng/ml; normal range, 0–20 ng/ml) concentrations, were not present. The patient was referred to the Department of Surgery (The 302nd Hospital) for further investigation of a hepatic tumor detected by a B-scan ultrasound.

The second case was of a 35-year-old male who was serologically positive for hepatitis B surface antigen and was admitted to the Department of Surgery with a history of persistent epigastric abdominal pain and weight loss. The serum concentrations of alkaline phosphatase and fibrinogen were increased to 259 U/l (normal range; 40–150 U/l) and 4.65g/l (normal range; 2.0–4.5 g/l), respectively. Serum α-fetoprotein (14.8 ng/ml; normal range, 0.00–20.00 ng/ml) concentrations were also normal. The patient underwent a liver ultrasonogram, which revealed a large hypoechoic lesion occupying the greater portion of the right lobe.

The two patients underwent MRI prior to therapy. MRI was performed using a 1.5-T MRI system (Signa HDx 1.5T; GE Healthcare, Cleveland, OH, USA). Overall, the imaging examinations performed consisted of axial T1 and T2 weighted imaging (WI) with fat suppression, in and out phase imaging, diffusion weighted imaging (b=0.800 sec/mm^2^) and use of post-contrast liver acquisition volume acceleration sequences, including arterial, portal venous, delayed phase and coronal T1WI with fat suppression.

**Figure 1:**
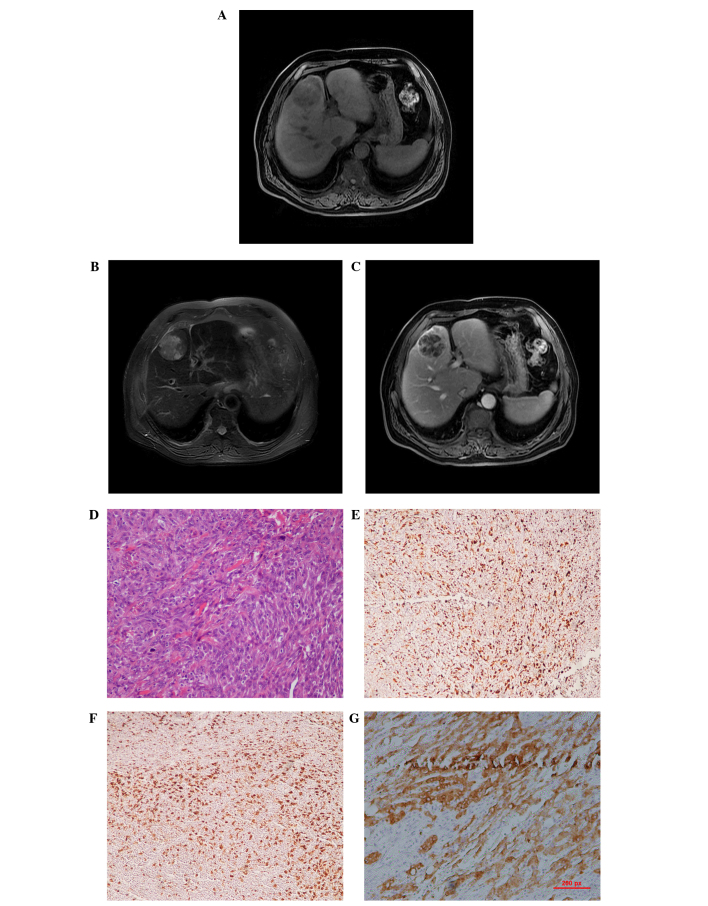
Case one: A 67-year-old male with primary malignant fibrous histiocytoma of the liver. (A) Magnetic resonance (MR) T1WI revealing a round-shaped mass, with hypointense areas in the tumor. (B) MR T2WI revealing a non-heterogeneous, hypointense lesion in the liver, with several discrete hyperintense areas in the central section of the tumor. (C) Contrast-enhanced T1WI revealing a large multilocular cystic mass, with a cystic wall, fibrous septa and enhancement of solid components. (D) Photomicrography of the round mass, consisting of closely packed spindle cells forming a storiform pattern (hematoxylin and eosin; ×100 magnification). (E–G) Tumor cells exhibiting immunoreactivity for vimentin (×100 magnification), cluster of differentiation 68 (×100 magnification) and cytokeratin 8 (×100 magnification), respectively. WI, weighted imaging.

The first case with MFH in the current study presented with a heterogeneous, low-attenuated lesion measuring 5.6×5.0×4.7 cm in the medial segment of the left lobe of the liver. The tumor was well-delineated from the surrounding liver parenchyma and the main signal intensity of the mass was hypointense compared with the liver, providing a result similar to that of skeletal muscle on T1WI. The main signal intensity of the mass on T2WI was high compared with that in the liver, and necrotic areas with a high signal intensity were found in the central tumor. The enhancement was mild and heterogeneous (Fig. 1A–C).

**Figure 2:**
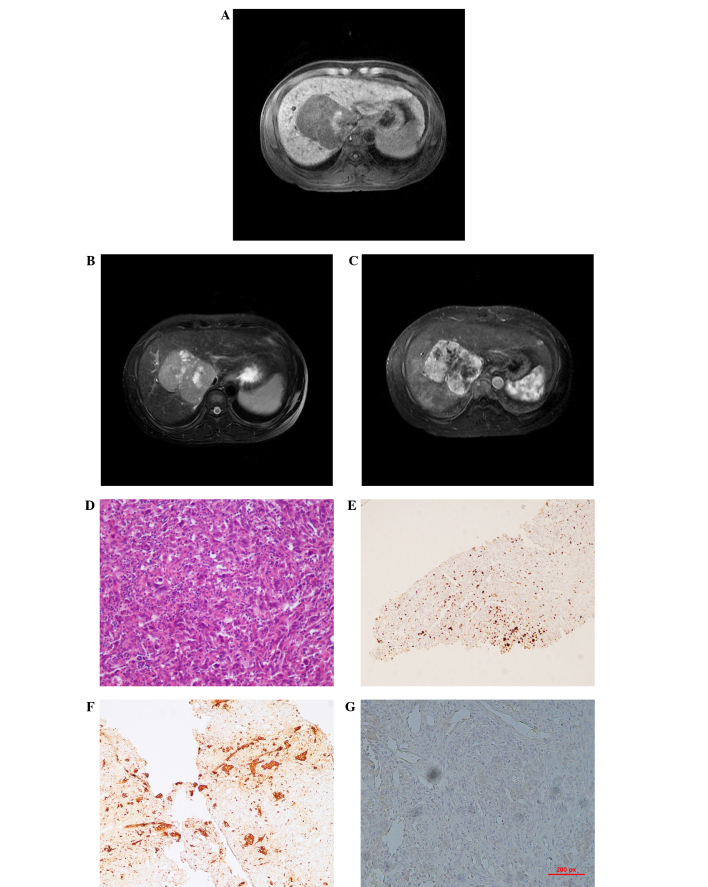
Case two: A 35-year-old male with liver cirrhosis due to hepatitis B virus infection and a tumor of the right lobe, confirmed to be a primary malignant fibrous histiocytoma of the liver. (A) The lesion is inhomogeneous on unenhanced T1WI and is mainly hypointense, with an irregular high intensity area in the left section of the mass. (B) The lesion possesses a well-defined margin and is inhomogeneously hyperintense on T2-weighted spin-echo magnetic resonance imaging, with numerous round- or oval-shaped patchy hyperintensity areas in the lesion. The intrahepatic biliary ducts are dilated. (C) Following IV administration of gadopenate dimeglumine, the heterogeneous enhancement of the tumor, with central hypointense sections, is clearly visible. (D) Hematoxylin and eosin staining of the pathological specimen (×100 magnification) reveals the spindle to consist of oval tumor cells arranged in a criss-cross fashion. (E–G) Immunohistological staining for vimentin (×100 magnification), cluster of differentiation 68 (×100 magnification) and for cytokeratin 8 (×100 magnification), respectively, demonstrating a positive reaction in the tumor cells. WI, weighted imaging.

Case two presented with a 10.9×8.9×7.1-cm tumor in the right lobe of the liver. This lesion had irregular geographical margins, with a minor bulge into the hepatic capsule. The tumor extended from the hepatic hilum to the liver bed and appeared to involve the gallbladder. The main signal intensity of the mass was hypointense on T1WI and was similar to the spleen. Multiple tiny foci of high signal intensity were visible on the post-contrast image, and marked enhancement and abundant blood vessels could be found surrounding the tumor mass (Fig. 2A–C). Neither patient had any other systemic tumors.

Pathological results were obtained by a hepatolobectomy in the first case and by a needle biopsy in the second case. Microscopically, the tumors of the two cases were similar and demonstrated proliferation of atypical cells, including spindle, pleomorphic and multi-nucleated giant cells arranged in storiform, sheet and/or fascicle patterns, with scattered foci of inflammatory cells, indicating the presence of MFH (Figs. 1D and 2D). Immunohistochemical examination revealed the tumors to be positive for vimentin, cluster of differentiation 68 and cytokeratin (CK)8 (Figs. 1E–G and 2E–G). Upon immunohistochemical staining of tissue from case one, the tumor was found to be negative for CK8 and vascular endothelial growth factor expression. A negative reaction for CK10 was revealed in the tissue from the second patient.

The post-operative period was uneventful in case one. However, the tumor of the second patient was unresectable and the patient was then referred to the oncology service for palliative chemotherapy (cisplatin, 75 mg/m^2^, d1–3 and actinomycin D, 60 mg/m^2^, d1). Following three cycles of chemotherapy the tumor increased in size. The patient then received cyroablation treatment, however the patient succumbed to a bleeding complication.

## Discussion

Malignant fibrous histiocytoma (MFH), considered to be a rarely occurring soft-tissue sarcoma of the liver in adults, has previously been defined as a pleomorphic malignant spindle cell neoplasm exhibiting fibroblastic and facultative histiocytic differentiation. However, the pre-operative diagnosis of hepatic MFH is made extremely challenging by the lack of clearly characteristic features. In order to diagnose primary hepatic MFH, two notable characteristics are necessary. The first involves the histopathology of the hepatic tumor. The second is that the clinicopathological criteria that define the tumor as hepatic in origin must be met. Li *et al* (5) proposed novel criteria for primary hepatic MFH in a review of seven cases. The criteria stated that the MFH must be a solitary or multifocal liver neoplasm without evidence of a pre-existing, co-existing or subsequently identified primary lesion at any location in the body. The essential point in establishing that the origin is hepatic is the exclusion of the possibility that the tumor is due to metastasis or direct invasion of MFH arising in other sites. Radiological imaging examinations and intraoperative gross examination at the time of the initial surgery revealed no additional tumors at any alternative site or organ in the present patients.

The imaging features of the tumors in the two cases were within the extent of the soft-tissue MFH classification. It has been reported that MFH appears as a well-defined mass that shows hypoechoic, mixed or hyperechoic patterns with variable anechoic areas. The complex internal pattern of MFH depends on the solid portion of high cellularity and the necrotic regions on the ultrasonogram (1). The features of hepatic MFH on unenhanced computed tomography vary and the scan may reveal a poorly-separated or well-delineated, large or multi-nodular mass, with a heterogeneous low attenuation density and numerous areas of necrosis. It has been reported that smaller tumors may prsent as a solid mass without prominent internal necrosis (4). However, in the present study, the smaller tumor exhibited an increased necrotic area compared with the larger tumor. Following contrast injection, the solid component demonstrated variable enhancement on delayed post-contrast scans, dependent on the tumor vascularity and the extent of the tumor necrosis.

MFH has been reported as inhomogeneously hyperintense on T2WI and inhomogeneously enhanced on gadobenate dimeglumine-enhanced T1-weighted gradient-recalled echo imaging (6,7), which is similar to the present case findings. In case two, cirrhosis was indicated by a diffuse nodular hyperintense signal on T1WI and a low-intensity nodule surrounded by high-intensity septa on T2WI. Hepatic MFH associated with advanced liver cirrhosis is extremely rare. Hwang *et al* reported a case of the simultaneous occurrence of MFH and hepatocellular carcinoma in a patient with a cirrhotic liver (8). Although the present study is too small to contribute any definitive diagnostic features of hepatic histiocytomas, the present cases exhibited variable and non-specific morphological findings on MRI. However, signs suggestive of malignancy were always present in the two patients, including heterogeneous intensity, necrotic areas and heterogeneous vascular enhancement.

MFH of the liver is generally recognized as having a high local recurrence rate and a significant metastasis rate. The risk of local recurrence and distant metastasis correlates with the depth and size of the primary tumor. Recurrence of the tumor is not uncommon, even when the resection margin is tumor-free (6). Distant metastases may spread via the circulatory (30%) and lymphatic (12%) systems (9).

The size and location of the tumor and the efficacy of the initial surgical removal all contribute markedly towards the prognosis of a patient with soft-tissue MFH (10). The tumor size is considered to be a clinically significant prognostic factor in hepatic MFH. Additionally, the histopathological grade is considered to be closely associated with the prognosis (11).

In conclusion, the present case study described two cases of rare MFH in the liver, which tended to appear as hepatocellular carcinoma. MRI of a primary liver MFH may reveal a well-encapsulated, inhomogeneously enhancing mass. Although MFH is a rare entity, the possibility of hepatic MFH should be acknowledged during differential diagnosis, even in cirrhotic patients, and hepatic spindle cell tumors require comprehensive tissue sampling and immunohistochemical analyses in order to be diagnosed.
